# Increased impact of the El Niño–Southern Oscillation on global vegetation under future warming environment

**DOI:** 10.1038/s41598-023-41590-8

**Published:** 2023-09-02

**Authors:** Thanh Le

**Affiliations:** 1 Department of Civil and Environmental Engineering, Sejong University, Seoul, 05006 Republic of Korea; 2 Present Address: School of Atmospheric Sciences, Nanjing University of Information Science and Technology, Nanjing, 210044 China

**Keywords:** Biogeochemistry, Climate sciences, Ecology, Environmental sciences

## Abstract

There are broad effects of vegetation changes on regional climate, carbon budget, the water cycle, and ecosystems’ productivity. Therefore, further knowledge of the drivers of future vegetation changes is critical to mitigate the influences of global warming. The El Niño–Southern Oscillation (ENSO) is a major mode of interannual climate variability and is likely to affect vegetation on the global scale. Nonetheless, little is known about the causal impacts of ENSO on future vegetation cover with changes in land use and a warming environment. Here, we examined the connections between ENSO and vegetation using leaf area index (LAI) data over the period 2015–2100 from Coupled Modeling Intercomparison Project Phase 6. Our findings indicate that, compared with the historical period 1915–2000, the vegetated areas influenced by ENSO are projected to rise by approximately 55.2% and 20.7% during the twenty-first century of the scenarios SSP2-4.5 and SSP5-8.5, respectively. Though uncertainty for the causal link between ENSO and vegetation changes remains in several regions (i.e., parts of North America, southern Australia, and western Asia), ENSO signature on LAI variations is robust over northern Australia, Amazonia, and parts of Southeast Asia. These results indicate that the influences of ENSO on global vegetation may strengthen in the future.

## Introduction

There are broad impacts of vegetation changes on the water cycle^1–5^, regional climate^6–12^, carbon budget^13–18^ and ecosystems productivity^19,20^. Hence, further understanding of the drivers of future vegetation changes is crucial to mitigate the impacts of global warming.

The El Niño–Southern Oscillation (ENSO), as a major mode of climate variability^21,22^, is expected to affect global vegetation changes. As the growth of trees depends on water availability^23^, ENSO-induced changes in the water cycle^24–26^ may lead to impacts on vegetation cover. For instance, ENSO shows an impact on global growing-season normalized difference vegetation index (NDVI_gs_)^27^, vegetation respiration^28^, vegetation health^29^, and plays an important role in altering the global crop production^30,31^. In particular, ENSO showed influences on tree-level ecophysiology via the impacts on regional water availability^32^ and ENSO-induced drought and soil drying may cause leaf shedding, tree mortality, and slow recovery of forests^33–35^. In addition, ENSO causes a shift in the response of leaf and seed fall over the tropical forest of Panama^36^, Amazonia^37^, and vegetation changes over eastern, southern, and western Africa^38–41^.

However, the causal impacts of ENSO on green vegetation cover in the future remain unclear. While long-term observed leaf area index (LAI) products are limited^17,42,43^, Earth system models provide important information on how forests may evolve with different scenarios of a future warming environment^23,44^. In addition, previous works focused on the correlation between ENSO and LAI, while causal analysis accounting for the confounding influence of other main climate modes is deficient.

In the present work, we evaluate the possibility of the causal impacts of ENSO on the global LAI using CMIP6 data over the 2015–2100 period. The outputs from CMIP6 models offer a valuable opportunity to assess the future influence of ENSO on vegetation cover.

## Data and methods

### Datasets

We used data from the historical simulation and ScenarioMIP^45,46^. We limited our study to two future scenarios SSP5-8.5 (i.e., Shared Socio‐Economic Pathway 5 and climate forcing level of 8.5 W/m^2^ at the year 2100) and SSP2-4.5 (i.e., Shared Socio‐Economic Pathway 2 and climate forcing level of 4.5 W/m^2^ at the year 2100). The scenario SSP5-8.5 represents the high end in the range of future forcing pathways while the scenario SSP2-4.5 represents the intermediate forcing level^46^. These future scenarios cover the 2015–2100 period. The historical simulation^45^ is utilized as a baseline to evaluate potential differences of the impacts of ENSO on the future global vegetation cover compared to the historical simulation. The historical simulation covers the period 1915–2000. Table S1 shows the 14 CMIP6 models which supplied vegetation cover data for the present work. The use of multiple model outputs reduces the uncertainty of the links between ENSO and green vegetation cover.

We used leaf area index (LAI) datasets from CMIP6 models. LAI (computed as one-sided green leaf area per unit ground surface) is an important indicator of vegetation cover or vegetation greenness and is a fundamental variable in land models^17^. LAI is also an important indicator of fuel supply, burning conditions, and fire prediction over a specific area^43,47^. The CMIP6 models may overestimate the mean LAI and the variations of LAI in some regions^48^. In addition, the models may have biases in simulating different phases (e.g., onset and length) of the growing season^49,50^ and the magnitude of LAI growth^51^. However, outputs from Earth system models are still helpful to investigate the variations of global vegetation cover^44,52,53^.

We utilized monthly sea level pressure (SLP) and sea surface temperature (SST) to compute the time series of major climate modes (see also Sect. "Methods" and Text S1).

### Methods

The methods used in this work are based on a multivariate predictive model^24,54^ to assess the null hypothesis of no Granger causality between ENSO and LAI. Our approach uses the *p*-value or probability value as a metric to estimate the likelihood for no causal effects of ENSO on LAI.

In the computations, we considered the confounding effects of other main climate modes (i.e., the Southern Annular Mode (SAM) (e.g., Cai et al.^55^), the Indian Ocean Dipole (IOD)^56,57^, and the North Atlantic Oscillation (NAO)^58^) on the connections between ENSO and LAI. Additional details of the methods are described in section Text S1.

## Results

### Model simulations of LAI

**Figure 1:**
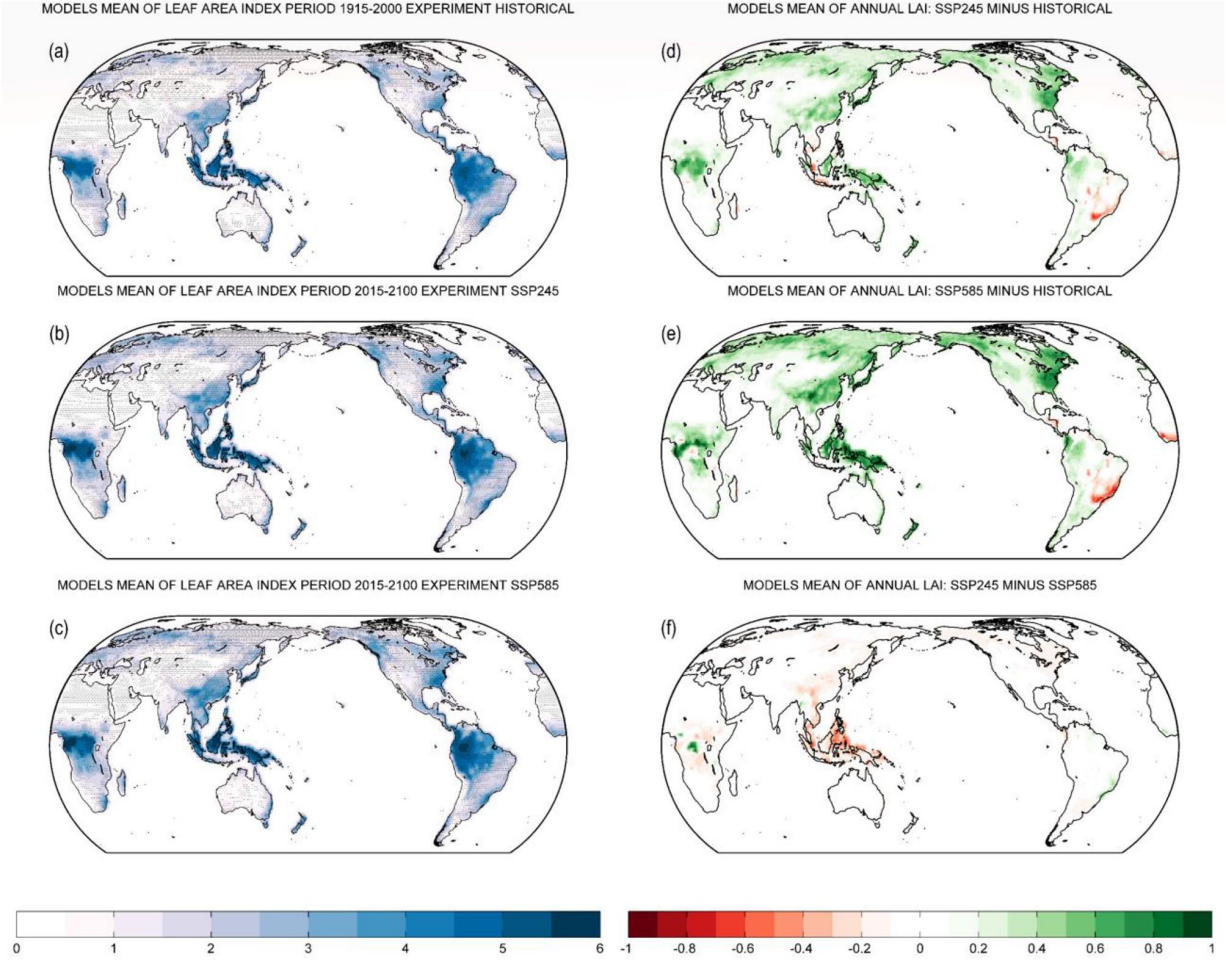
(**a**–**c**) Multi-model mean map of annual LAI (m^2^ m^-2^) over the 1915–2000 period of the historical experiment (**a**) and over the 2015–2100 period of the future scenarios SSP2-4.5 (**b**) and SSP5-8.5 (**c**). In (**a**), (**b**) and (**c**), stippling implies that at least 70% of total models show agreement on the mean LAI of all models at a given grid point. The agreement of a single model is defined when the difference between the selected model's LAI and the multi-model mean LAI is less than one standard deviation of the multi-model mean LAI. Blue shades imply a high annual LAI. (**d**) Difference of multi-model mean annual LAI between future scenario SSP2-4.5 and historical experiment (i.e., SSP2-4.5 minus historical experiment). (**e**) As in (**d**), but for future scenario SSP5-8.5 and historical experiment (i.e., SSP5-8.5 minus historical experiment). (**f**) As in (**d**), but for future scenario SSP2-4.5 and future scenario SSP5-8.5 (i.e., SSP2-4.5 minus SSP5-8.5. LAI: Leaf Area Index.

The models mean map of LAI is shown in Fig. 1a–c for the historical experiment and two future scenarios SSP2-4.5 and SSP5-8.5, respectively. There is a high agreement between the models for most vegetated areas (denoted by stippling in Fig. 1a–c) in simulating LAI trends in the past and the future. The multi-model standard deviation of LAI for each simulation is depicted in Fig. S1. In the projections, there is an increase in the global LAI compared to the historical experiment (Fig. 1d, e). The largest increases in LAI are over middle Africa, southeast Asia, east Asia, Alaska, and eastern North America. LAI is projected to decrease over a few spots over western tropical Africa, Central America, eastern South America, and part of southeast Asia. The largest decrease in LAI is observed over eastern South America in SSP5-8.5 scenario (Fig. 1e). The higher increase in LAI for most regions is observed for SSP5-8.5 scenario compared to SSP2-4.5 scenario (Fig. 1f).

**Figure 2:**
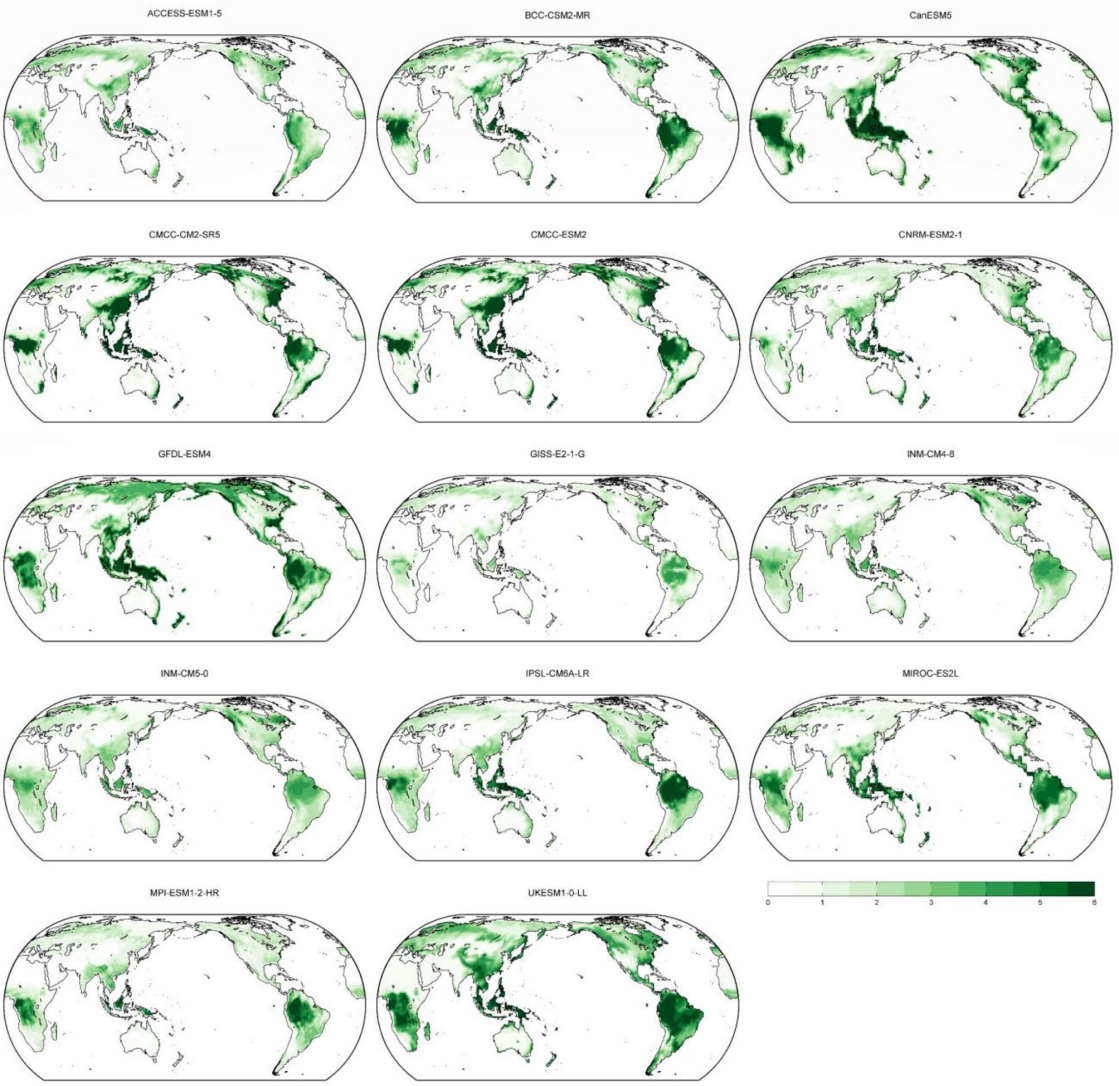
Map of annual LAI (m^2^ m^-2^) over the period 2015–2100 for the future scenario SSP5-8.5 of 14 single models (see Table S1). LAI: Leaf Area Index.

Figure 2 describes the global LAI of 14 single models (see also Table S1). The spatial pattern of global LAI is largely consistent for most models with higher LAI observed over the tropics, east Asia, parts of Europe, and parts of North America. Several models (e.g., ACCESS_ESM1_5, GISS-E2-1-G) show weaker simulated LAI compared to the models mean and other models.

### ENSO causal impacts on annual mean LAI

**Figure 3:**
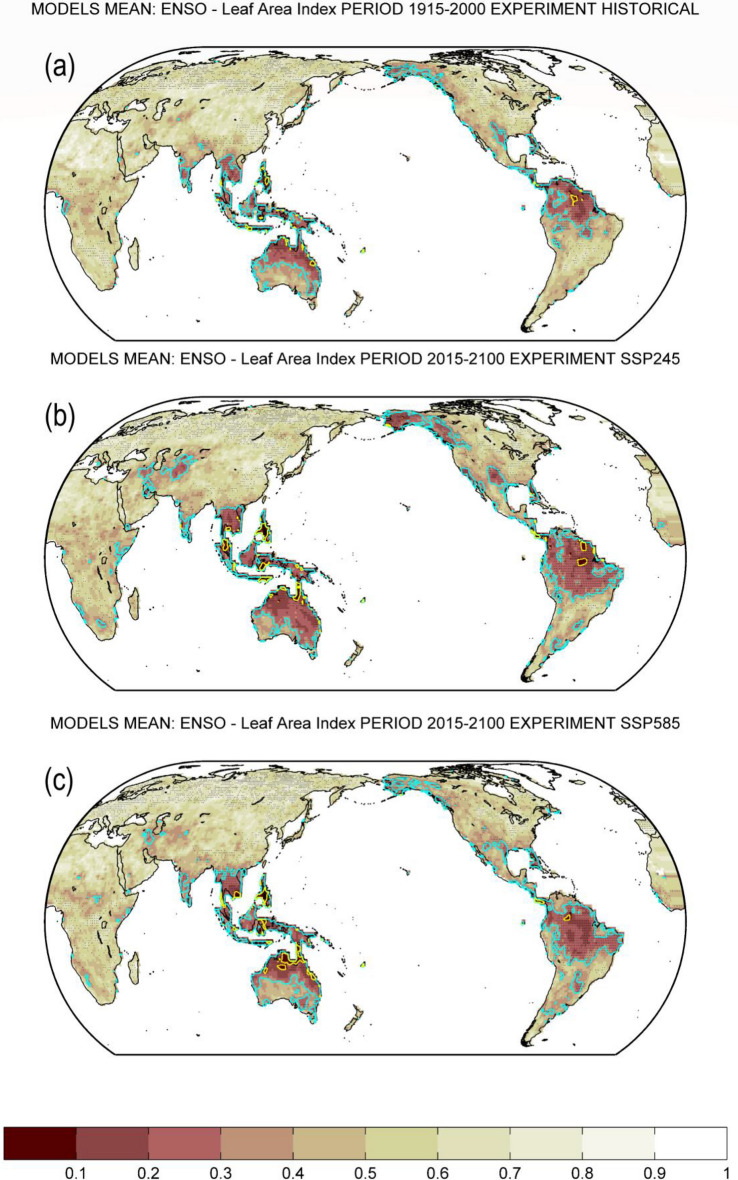
Map of multi-model mean probability for no Granger causality from ENSO [D(*t*)JF(*t* + *1*)] to annual mean LAI over the period 1915–2000 of the historical simulation (**a**) and over the period 2015–2100 of the future scenarios SSP2-4.5 (**b**) and SSP5-8.5 (**c**). Stippling implies that at least 70% of total models show agreement on the mean probability of all models at a given grid point. The agreement of a single model is given when the difference between the selected model's probability and the multi-model mean probability is less than one standard deviation of the multi-model mean probability. In (**a**), (**b**) and (**c**), the cyan and yellow contour lines indicate *p*-value = 0.33 and 0.1, respectively. Brown shades denote a low probability for no Granger causality. ENSO: El Niño–Southern Oscillation. LAI: Leaf Area Index.

Figure 3 illustrates the multi-model mean of the causal effects of ENSO on global LAI for the historical experiment over the 1915–2000 period (a) and the projections over the 2015–2100 period of the two future scenarios SSP2-4.5 (b) and SSP5-8.5 (c). We show that ENSO is unlikely (very unlikely) to exhibit no causal effects on LAI (i.e., *p*-value are lower than 0.33 (0.1)) over the tropics, western and southern North America, Australia, southern South America, and Central Asia.

ENSO impacts on LAI are not seen over central Asia in historical periods (Fig. 3a), however, these impacts appear to be more significant in the future projections (Fig. 3b, c). The effects of ENSO on LAI are generally weak over Africa, except few spots over eastern and southern Africa. There is a high model consensus (denoted by stippling in Fig. 3) of ENSO effects on LAI over parts of Southeast Asia, northern South America which includes Amazonia, and parts of northwestern North America.

**Figure 4:**
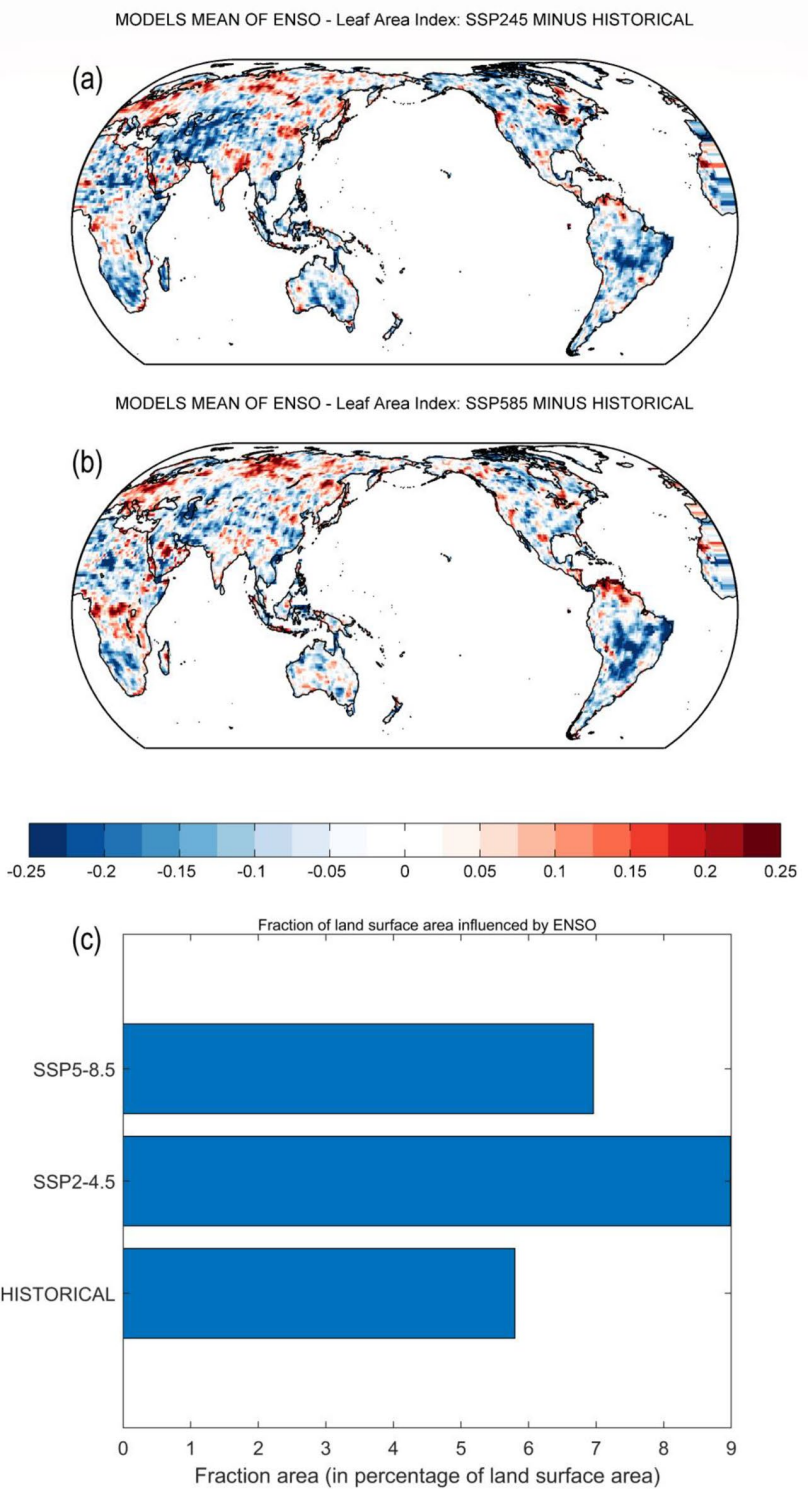
(**a**) Difference of multi-model mean probability for no Granger causality from ENSO [D(*t*)JF(*t* + *1*)] to annual mean LAI between future scenario SSP2-4.5 and historical experiment (i.e., SSP2-4.5 minus historical experiment). (**b**) As in (**a**), but for future scenario SSP5-8.5 and historical experiment (i.e., SSP5-8.5 minus historical experiment). In (**a**) and (**b**), red (blue) shades imply a higher (lower) probability for the absence of Granger causality in the future scenarios SSP2-4.5 and SSP5-8.5 compared to the historical experiment. (**c**) Fraction of total land-area with probability for the absence of Granger causality from ENSO to LAI lower than 0.33 (i.e., *p*-value < 0.33). Fraction areas are presented for the historical experiment, the future scenarios SSP2-4.5 and SSP5-8.5. ENSO: El Niño–Southern Oscillation. LAI: Leaf Area Index.

Figure 4 reveals the differences between historical and future simulated patterns of ENSO impacts on LAI. We observed an expansion of ENSO impacts over the tropics, Central Asia, and North America (Fig. 4a, b). These increases in ENSO impacts are associated with the increase in LAI in some regions (Fig. 1). On the opposite, there is a decline in the likelihood of ENSO effects over tropical Africa, northern South America, and part of western North America (only for SSP5-8.5 scenario). Figure 4c suggests that the regions with considerable ENSO effects on LAI account for approximately 7% of global land-area in the SSP5-8.5 scenario and approximately 9% of global land-area in the SSP2-4.5 scenario. The areas impacted by ENSO in the historical period account for approximately 5.8% of global land-area. Compared to the historical period, there is a significant increase of around 55.2% and 20.7% of the affected land area in the scenarios SSP2-4.5 and SSP5-8.5, respectively. The increase in ENSO impacts in the SSP2-4.5 scenario is mainly found over western North America, Australia, and northern South America, while the increase in the SSP5-8.5 scenario is limited to northern South America (Fig. 3).

**Figure 5:**
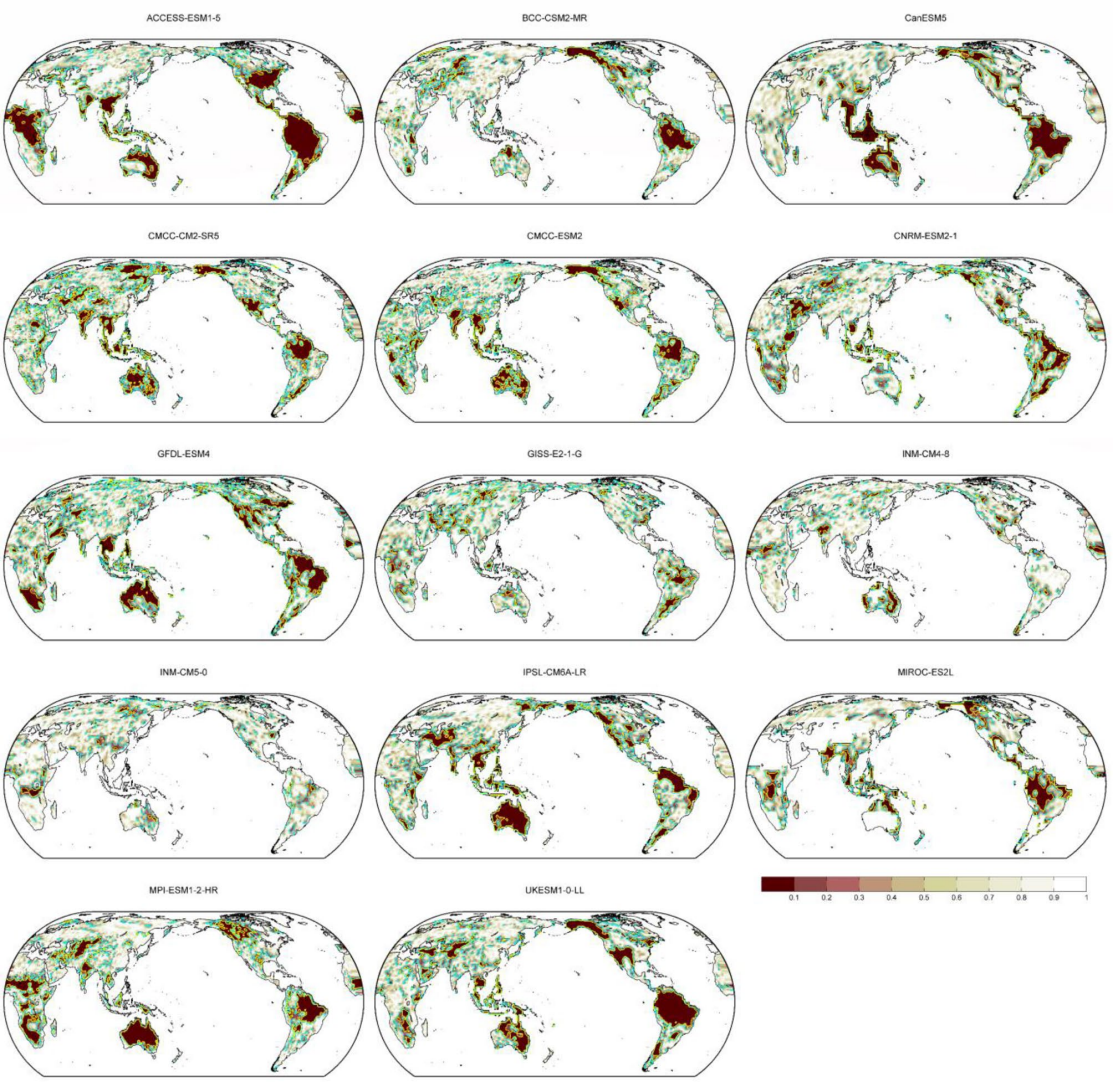
Map of probability for no Granger causality from ENSO [D(*t*)JF(*t* + *1*)] to annual mean LAI over the period 2015–2100 of the future scenario SSP2-4.5 for 14 single models (see Table S1). Stippling implies that at least 70% of total models show agreement on the mean probability of all models at a given grid point. The agreement of a single model is given when the difference between the selected model's probability and the multi-model mean probability is less than one standard deviation of the multi-model mean probability. The cyan and yellow contour lines indicate *p*-value = 0.33 and 0.1, respectively. Brown shades denote a low probability for no Granger causality. ENSO: El Niño–Southern Oscillation. LAI: Leaf Area Index.

Figure 5 shows the outcomes of 14 single models (see also Table S1) for the causal impacts of ENSO on LAI over the 2015–2100 period of the future scenario SSP2-4.5 (i.e., with stronger ENSO global impacts compared to the SSP5-8.5 scenario as shown in Figs. 3 and 4). In Fig. 5, several models (i.e., BCC_CSM2_MR, GISS-E2-1-G, INM-CM4-8, and INM-CM5-0) underestimate the response of vegetation to ENSO over tropical southeast Asia and Australia compared to the models’ mean. The models ACCESS_ESM1_5 and MPI_ESM1_2_HR show a stronger effect of ENSO on vegetation over Africa compared to other models. The spread of models in simulating the response of vegetation to ENSO may imply biases in the interactions between land, air, and biosphere in the models.

### ENSO causal impacts on seasonal mean LAI

**Figure 6:**
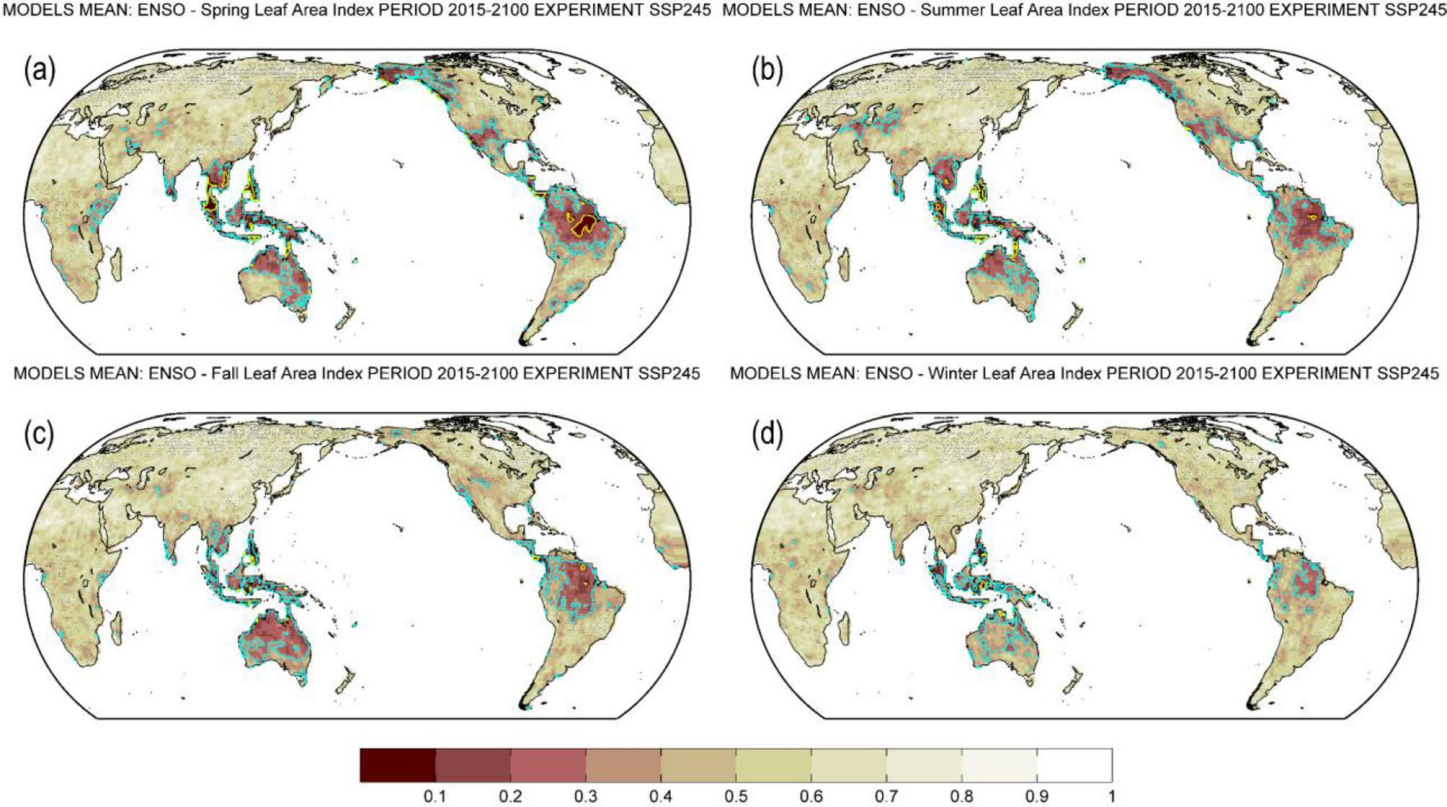
Map of multi-model mean probability for no Granger causality from ENSO during boreal winter [defined as D(*t*)JF(*t* + *1*); *t* denotes year *t*] to seasonal mean LAI over the 2015–2100 period of the future scenario SSP2-4.5. (**a**) Spring [March, April, May; MAM(*t* + *1*)]. (**b**) Summer [June, July, August; JJA(*t* + *1*)]. (**c**) Fall [September, October, November; SON(*t* + *1*)]. (**d**) Winter [December, January, February; D(*t* + *1*)JF(*t* + *2*)]. Stippling denotes that at least 70% of total models show agreement on the mean probability of all models at a given grid point. The cyan and yellow contour lines imply *p*-value = 0.33 and 0.1, respectively. Brown shades denote a low probability for no Granger causality. ENSO: El Niño–Southern Oscillation. LAI: Leaf Area Index.

Figure 6 shows the causal influences of ENSO (D(*t*)JF(*t* + *1*), see also text S1 for definitions) on seasonal LAI (text S1). In Fig. 6, ENSO impacts on seasonal LAI are significant in all four seasons over much of the tropics. ENSO impacts over Australia are expanded in the following boreal fall (SON(*t* + *1*)), the growing season in the southern hemisphere (Fig. 6c). The response of seasonal LAI to ENSO over North America weakens from boreal fall. The impacts of ENSO on vegetation are fading in the next winter (D(*t* + *1*)JF(*t* + *2*)) for most regions, except the tropics (Fig. 6d). ENSO impacts over central Asia are mainly apparent in the following boreal summer (JJA(*t* + *1*)) as shown in Fig. 6b. The vegetated areas affected by ENSO are largest in the following boreal summer and spring, accounting for approximately 7.1% and 6.8% of the land-area, respectively (Fig. S2). In the following boreal fall and winter, this area reduces to approximately 5.9% and 2.4% of the land-area, respectively (Fig. S2).

## Discussion and conclusions

The increase in the global LAI in the future scenarios (2015–2100) compared to the historical period (1915–2000) (Fig. 1) is contributed by a warming environment, an increase in atmospheric CO_2_ and nitrogen deposition, and changes in land use and land cover^43,59–62^ with CO_2_ fertilization is the primary driver^17^. This increase in the global LAI is consistent with the greening trends reported in recent years^3,17,62–65^ and these trends may continue in the future^66^. While land cover change mainly contributed to the regional greening observed in the eastern United States and southeast China^62^, the decrease in LAI over eastern South America (Fig. 1) might be associated with the drying trend in recent years, driven by both changes in climate and land management^67^.

Enhanced ENSO impacts on LAI (Figs. 3 and 4) might be associated with land-use change and regrowth of vegetation in some areas^14^ and an increase in ENSO variability and ENSO-induced atmospheric teleconnections^68–71^. Significant impacts of ENSO on vegetation over Southeast Asia, Australia, and South America are consistent with recent works^24,26,72^ which showed a strong ENSO signature on the regional water cycle. ENSO impacts on a few spots over eastern and western Africa show an agreement with recent studies^38–40^. Increase of ENSO impacts over Northern America and central Asia might be related to warmer temperature and reforestation or afforestation over the extratropic^63,73^. These results highlight the important role of ENSO in the future change of global vegetation.

The vegetation area affected by ENSO might be low compared to previous studies^27,29^ using correlation analysis. The higher influence of ENSO is related to the confounding factors (e.g., the SAM, the IOD and the NAO) which are not considered in the correlation analysis. Different land-use scenarios may influence the connection between ENSO and LAI. For example, the land use changes employed in SSP5-8.5 are more extreme compared to SSP2-4.5 (i.e., the global time series of pastureland area are lower in SSP5-8.5)^46,74^. These differences in land-use scenarios may lead to more significant impacts of ENSO on LAI for SSP2-4.5 compared to SSP5-8.5.

The high agreement of the models in simulating past and future LAI (Figs. 1 and 2) suggests that the models may have the capability to project future LAI trend and outputs from CMIP6 models are useful. Despite high consistency in simulating global LAI (Figs. 1 and 2), some models’ discrepancy of ENSO effects is observed in several regions (Figs. 3 and 5), suggesting that improvement in the models may benefit further understanding of ENSO impacts on future global vegetation.

### Supplementary Information

Supplementary Information.

## Data Availability

The data that support the findings of this study are openly available at the following website: https://esgf-node.llnl.gov/search/cmip6/.
