# Supplementary material for: Increased impact of the El Niño–Southern Oscillation on global vegetation under future warming environment
Source: Sci Rep. 2023 Sep 2;13:14459. doi: 10.1038/s41598-023-41590-8 (PMC10475042; doi:10.1038/s41598-023-41590-8)
Supplement: Supplementary file 1 — Supplementary Information. [file 41598_2023_41590_MOESM1_ESM.docx]

Supporting Information for

**Increased impact of the El Niño–Southern Oscillation on global vegetation under future warming environment**

Thanh Le^1🟉^

^1^Department of Civil and Environmental Engineering, Sejong University, Seoul 05006, Republic of Korea

^🟉^Corresponding author(s): Thanh Le ([thanhle@sejong.ac.kr](mailto:thanhle@sejong.ac.kr))

**Contents of this Supporting Information**

Text S1

Table S1

Figures S1 to S2

# Text S1

We use the following multivariate predictive model ^1,2^ to estimate the causal links between the ENSO and leaf area index or content:

$\boldsymbol{X}_{\boldsymbol{t}}\boldsymbol{=}\sum_{\boldsymbol{i}\boldsymbol{=}\boldsymbol{1}}^{\boldsymbol{p}} \boldsymbol{\alpha}_{\boldsymbol{i}}\boldsymbol{X}_{\boldsymbol{t}\boldsymbol{-}\boldsymbol{i}}\boldsymbol{+}\sum_{\boldsymbol{i}\boldsymbol{=}\boldsymbol{1}}^{\boldsymbol{p}} \boldsymbol{\beta}_{\boldsymbol{i}}\boldsymbol{Y}_{\boldsymbol{t}\boldsymbol{-}\boldsymbol{i}}\boldsymbol{+}\sum_{\boldsymbol{j}\boldsymbol{=}\boldsymbol{1}}^{\boldsymbol{m}} \sum_{\boldsymbol{i}\boldsymbol{=}\boldsymbol{1}}^{\boldsymbol{p}} \boldsymbol{\delta}_{\boldsymbol{j}\boldsymbol{,}\boldsymbol{i}}\boldsymbol{Z}_{\boldsymbol{j}\boldsymbol{,}\boldsymbol{t}\boldsymbol{-}\boldsymbol{i}}\boldsymbol{+}\boldsymbol{\varepsilon}_{\boldsymbol{t}}$ (1)

where *X_t_* is the annual mean (or seasonal mean) leaf area index for year *t*, *Y_t_* is the ENSO index, and *Z_j,t_* is the confounding factor *j* for year *t*. In the predictive model shown in equation 1, while estimating the influence of $Y$ on $X$ (i.e., the contribution of the term $\sum_{i=1}^{p} \beta_{i}Y_{t-i}$in predicting $X$), the contribution of past $X$ events are already taken into account by adding the term $\sum_{i=1}^{p} \alpha_{i}X_{t-i}$. Thus, the causal influence of $Y$ on $X$, if detected, is robust and the contribution of past $X$ events are already considered in our analyses. Here, *m* is number of confounding factors and *p* ≥ 1 is the order of the multivariate predictive model. The optimal order *p* is computed by minimizing the Schwarz criterion or the Bayesian information criterion. The optimal orders might be different for each model.

The ENSO index was computed as the average sea surface temperature (SST) anomalies in the Niño 3.4 area (120–170°W; 5°N–5°S) in boreal winter (December–January–February, DJF). Confounding factors (i.e., the dipole mode index (DMI; Saji et al., 1999), the Southern Annular Mode (SAM) and the North Atlantic Oscillation (NAO; e.g., Hurrell et al., 2003)) may have effects on the connections between ENSO and leaf area index. The DMI was given as the difference in boreal fall (September–October–November, SON) SST anomalies between two Indian Ocean regions of the western pole (50–70°E; 10°N–10°S) and southeastern pole (90–110°E; 0°N–10°S). The SAM ^5^ was calculated as the first empirical orthogonal function (EOF) of the boreal summer (June–July–August, JJA) sea level pressure (SLP) anomalies for the region of 40–70°S. The NAO index is computed as the EOF of boreal winter (DJF) SLP anomalies in the North Atlantic area (90ºW-40ºE, 20º-70ºN).

Here we consider the simultaneous impacts of confounding factors and thus provide more information of the real-world teleconnections. Our analysis uses three different confounding factors; thus, *m* is equal to 3. The noise residuals *ε_t_* and the regression coefficients *α_i_*, *β_i_* and *δ_j,i_* are the results of the multiple linear regression analysis using the least squares method. We detrend and normalize all the climate indices.

We estimate the probability of no Granger causality by applying a test of Granger causality ^1,2^ for the multivariate predictive model shown in equation 1.

For computing the degree of uncertainty, we followed recent guidance ^6^ and utilized the terms ‘very unlikely’, ‘unlikely’, ‘likely’ for the 0–10%, 0–33%, and 66–100% probability of the likelihood of the outcome, respectively. For example, if the *p*-value is less than 0.33, the result indicates that ENSO is unlikely to display no Granger causality on LAI. In this instance, we conclude that ENSO has ‘causal effect’ on LAI.

Regarding the connection between ENSO and seasonal leaf area index, we examine the causal impacts of ENSO in boreal winter of year *t* (defined as [D(*t*)JF(*t+1*)]) on the LAI in the following four periods of years *t+1* and *t+2* with three months each period. The three periods from March to May, from June to August, and from September to November in year *t+1* are defined as [MAM(*t+1*)], [JJA(*t+1*)], [SON(*t+1*)], respectively. The next period just after [SON(*t+1*)] in years *t+1* and *t+2* is defined as [D(*t+1*)JF(*t+2*)].

# References

1. Stern, D. I. & Kaufmann, R. K. Anthropogenic and natural causes of climate change. *Clim. Change* **122**, 257–269 (2013).

2. Mosedale, T. J., Stephenson, D. B., Collins, M. & Mills, T. C. Granger Causality of Coupled Climate Processes: Ocean Feedback on the North Atlantic Oscillation. *J. Clim.* **19**, 1182–1194 (2006).

3. Saji, N. H., Goswami, B. N., Vinayachandran, P. N. & Yamagata, T. A dipole mode in the tropical Indian Ocean. *Nature* **401**, 360–363 (1999).

4. Hurrell, J. W., Kushnir, Y., Ottersen, G. & Visbeck, M. An overview of the North Atlantic Oscillation. in *Geophysical Monograph American Geophysical Union* 1–35 (American Geophysical Union, 2003). doi:10.1029/134GM01.

5. Cai, W., Sullivan, A. & Cowan, T. Interactions of ENSO, the IOD, and the SAM in CMIP3 Models. *J. Clim.* **24**, 1688–1704 (2011).

6. Stocker, T. F. *et al.* Technical Summary. in *Climate Change 2013 - The Physical Science Basis* (ed. Intergovernmental Panel on Climate Change) 31–116 (Cambridge University Press, 2013). doi:10.1017/CBO9781107415324.005.

Table S1. List of CMIP6 models used in this study.

| No. | Model name | Modelling center, country | Land model name |
| --- | --- | --- | --- |
| 1 | ACCESS_ESM1_5 | CSIRO, Australia | CABLE2.5 |
| 2 | BCC_CSM2_MR | BCC, China | BCC_AVIM2 |
| 3 | CanESM5 | CCCma, Canada | CLASS3.6/CTEM1.2 |
| 4 | CMCC-CM2-SR5 | CMCC, Italy | CLM4.5 (BGC mode) |
| 5 | CMCC-ESM2 | CMCC, Italy | CLM4.5 (BGC mode) |
| 6 | CNRM_ESM2_1 | CNRM-CERFACS, France | Surfex 8.0c |
| 7 | GFDL_ESM4 | NOAA-GFDL, United States | GFDL-LM4.1 |
| 8 | GISS-E2-1-G | NASA-GISS, United States | GISS LSM |
| 9 | INM-CM4-8 | INM, Russia | INM-LND1 |
| 10 | INM-CM5-0 | INM, Russia | INM-LND1 |
| 11 | IPSL-CM6A-LR | IPSL, France | ORCHIDEE (v2.0) |
| 12 | MIROC-ES2L | MIROC, Japan | MATSIRO6.0+VISIT-e ver.1.0 |
| 13 | MPI_ESM1_2_HR | MPI-M, Germany | JSBACH3.20 |
| 14 | UKESM1_0_LL | MOHC NERC, United Kingdom | JULES-ES-1.0 |


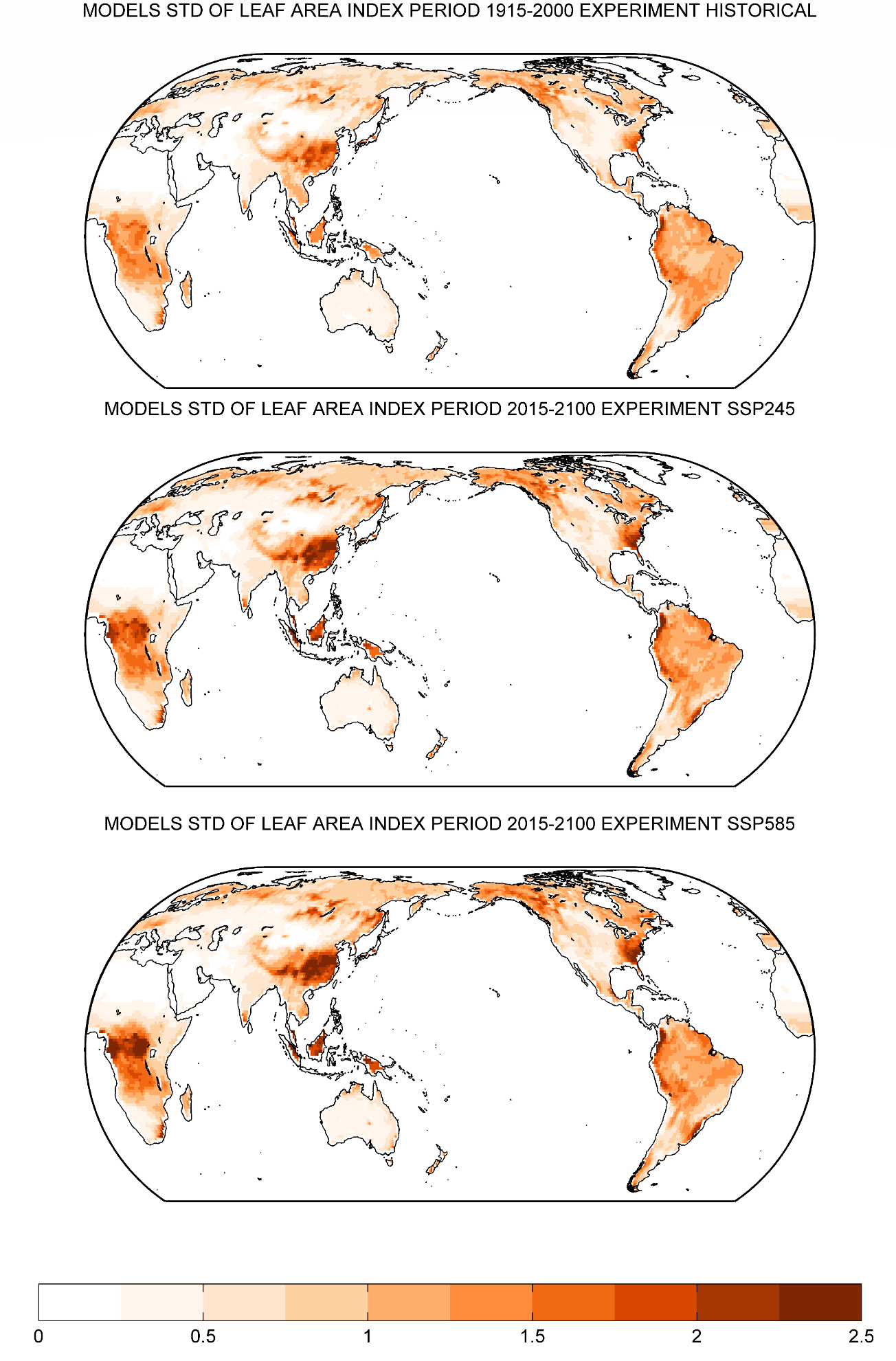


Figure S1. Multi-model map of standard deviation of annual LAI (m^2^ m^-2^) over the period 1915-2000 of the historical simulation (a) and over the period 2015-2100 of the future scenarios SSP2-4.5 (b) and SSP5-8.5 (c). LAI: Leaf Area Index.


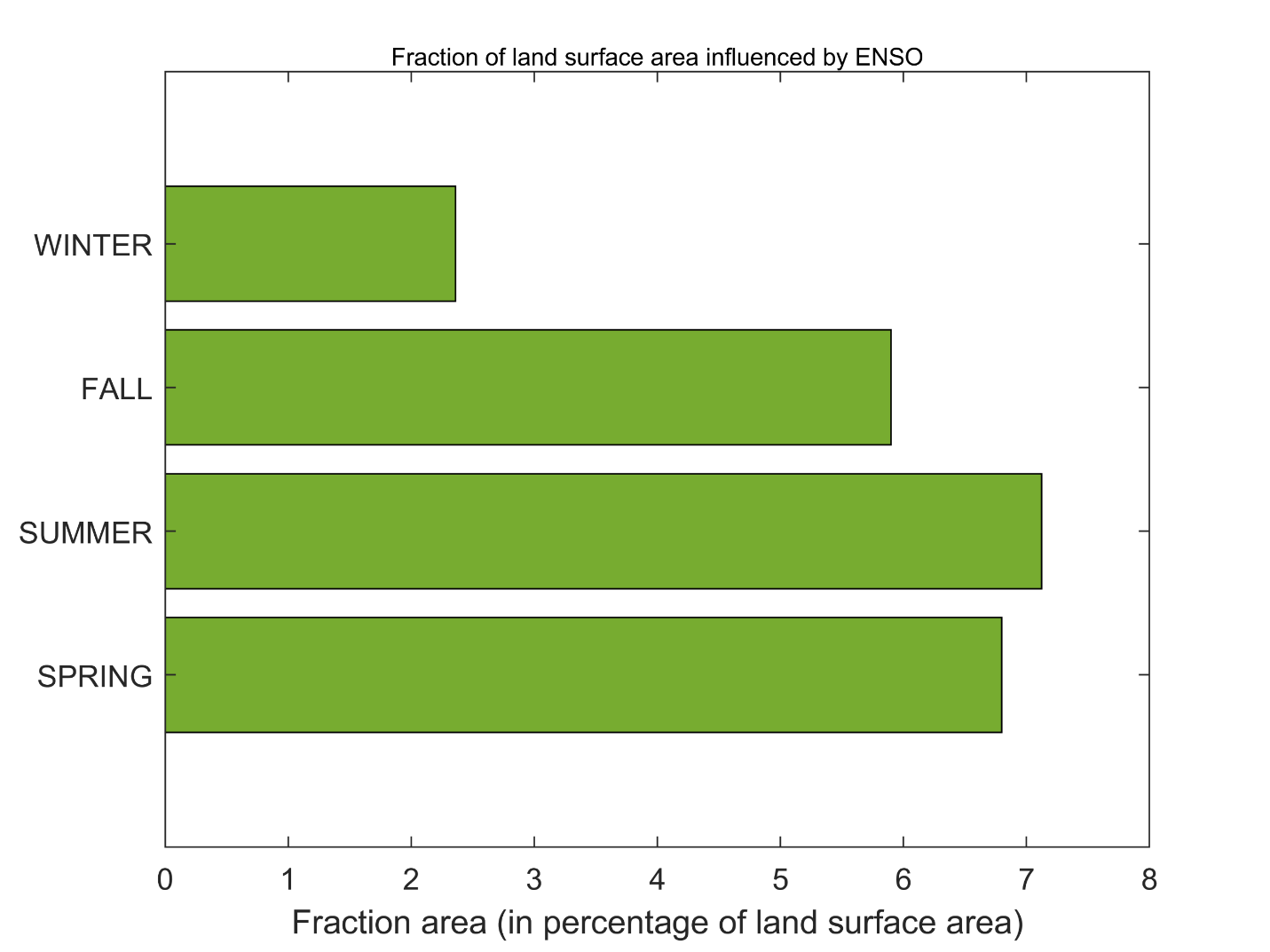


Figure S2. Fraction of land surface with probability for the absence of Granger causality from ENSO [D(*t*)JF(*t+1*)] to seasonal LAI lower than 0.33 (i.e., *p*-value < 0.33). The results are shown for the future scenario SSP2-4.5 over the 2015-2100 period. Fraction areas influenced by ENSO on spring [MAM(*t+1*)], summer [JJA(*t+1*)], fall [SON(*t+1*)], winter [D(*t+1*)JF(*t+2*)] surface carbon cycle are presented. ENSO: El Niño–Southern Oscillation. LAI: Leaf Area Index.
